# Supplementary material for: Ufbp1 promotes plasma cell development and ER expansion by modulating distinct branches of UPR
Source: Nat Commun. 2019 Mar 6;10:1084. doi: 10.1038/s41467-019-08908-5 (PMC6403283; doi:10.1038/s41467-019-08908-5)
Supplement: Supplementary file 1 — Supplementary Information [file 41467_2019_8908_MOESM1_ESM.pdf]

Ufbp1 promotes plasma cell development and ER expansion by modulating distinct branches of UPR

Zhu et al.

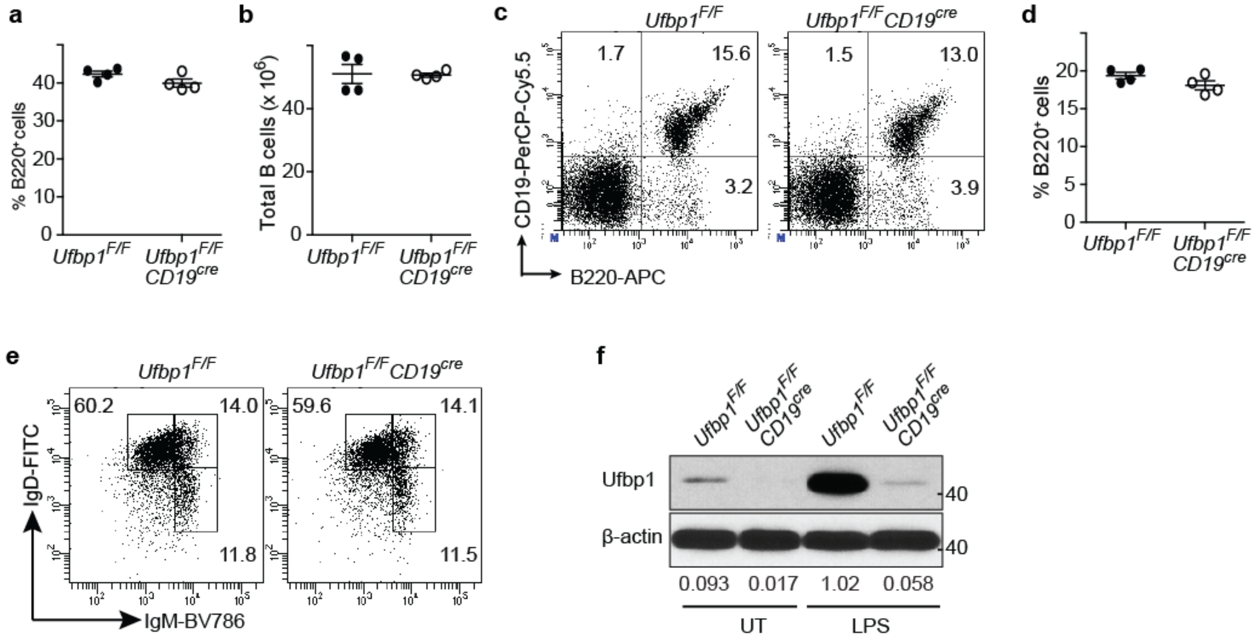

Supplementary Figure 1. Normal development of B cells in *Ufbp1$^{F/F}$CD19^{cre}* mice.
a, Enumeration of the frequencies of B220^{+} cells in Fig. 1a. **b,** total numbers of B220^{+} cells in spleens of *Ufbp1$^{F/F}$* and *Ufbp1$^{F/F}$CD19^{cre}* mice (n=4 mice/genotype). **c,** Expression of B220 and CD19 by lymph node cells of *Ufbp1$^{F/F}$* and *Ufbp1$^{F/F}$CD19^{cre}* mice. **d,** Enumeration of the frequency of B220^{+} cells in c (n=4 mice/genotype). **e,** IgM and IgD expression by B220^{+} splenic cells from *Ufbp1$^{F/F}$* and *Ufbp1$^{F/F}$CD19^{cre}* mice. **f,** Total cell lysates from B cells untreated or stimulated with LPS for three days from *Ufbp1$^{F/F}$* and *Ufbp1$^{F/F}$CD19^{cre}* mice were immunoblotted with antibodies against Ufbp1 and β-actin. The numbers indicate the ratio of intensity of Ufbp1 band to the corresponding β-actin band. Error bars represent mean ± SE. A representative of at least two experiments is shown.

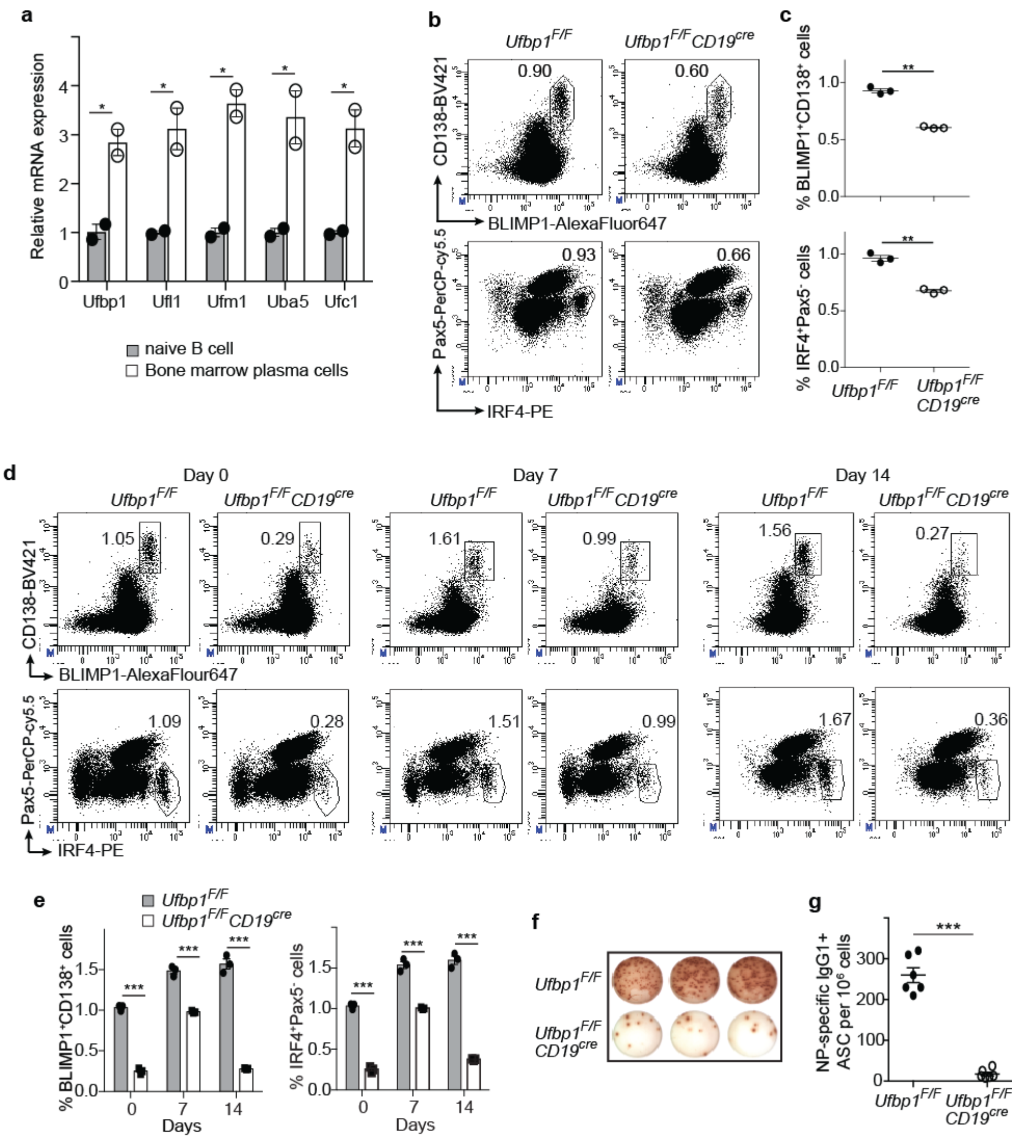

Supplementary Figure 2. Ufbp1 regulates of plasma cells. a, Expression of Ufbp1, Ufl1, Ufm1, Uba5, and Ufc1 mRNA in naïve B cells ($B220^+CD138^-$) and bone marrow plasma cells ($B220^-CD138^+$) was quantified by qRT-PCR. **b,** Spleen cells from indicated mice were stained with BLIMP1, CD138, IRF4 and Pax5, and analyzed by flow cytometry. Upper and lower panels show staining for BLIMP1 versus CD138, and IRF4 versus Pax5 respectively. **c,** Enumeration of plasma cells ($BLIMP1^+CD138^+$ and $IRF4^+Pax5^-$) frequencies in b (n=3 mice/genotype). **d,** Flow cytometric detection of plasma cells ($BLIMP1^+CD138^+$, and $IRF4^+Pax5^-$ cells) in the spleens of *Ufbp1*$^{F/F}$ and *Ufbp1*$^{F/F}$*CD19*^{cre} mice at indicated time points following immunization with NP-KLH in Fig 2c. **e,** Enumeration of the frequencies of plasma cells ($BLIMP1^+CD138^+$, and $IRF4^+Pax5^-$) in d, (n=3 mice/genotype). **f,** Splenocytes from mice in Fig 2e were analyzed for presence of NP-specific $IgG1^+$ASCs by ELISpot. Shown is a photograph of representative ELISpot wells. **g,** Frequencies of NP-specific $IgG1^+$ ELISpots in f. Error bars represent mean ± SE. * $P < 0.05$, ** $P < 0.01$, *** $P < 0.001$. Unpaired student's two-tailed t-test was used. A representative of two experiments is shown.

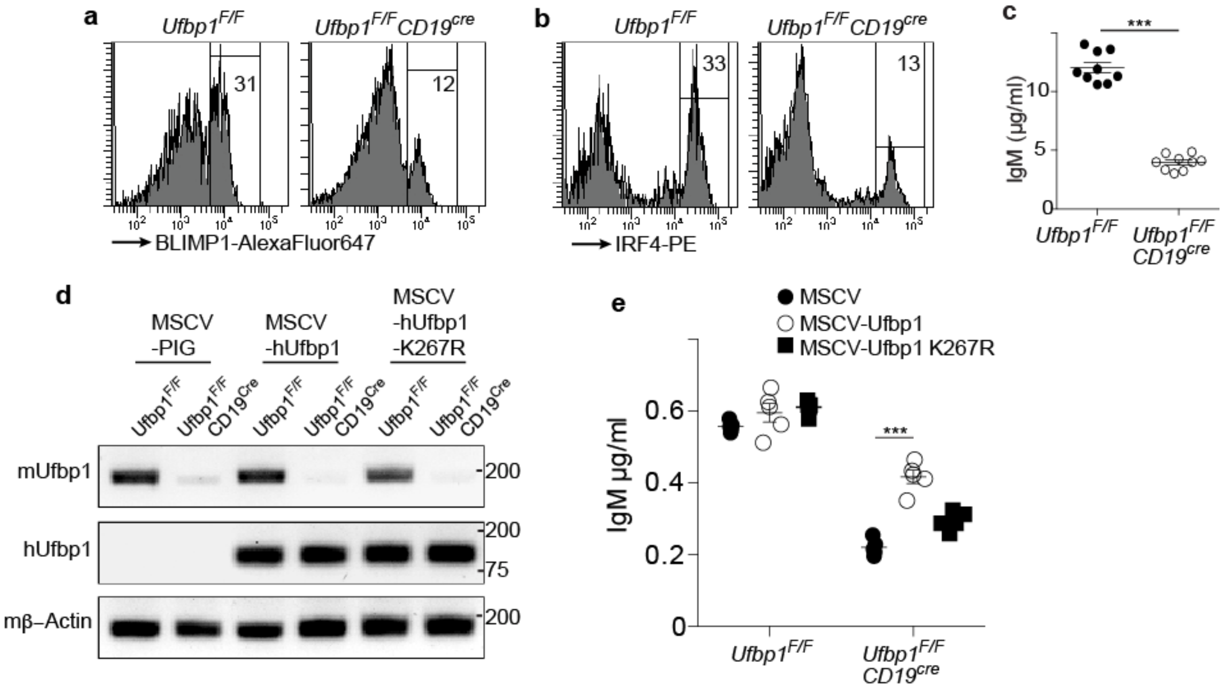

Supplementary Figure 3. A role for Ufbp1 in expression of BLIMP1 and IRF4.
a and b, Data from Fig 3a and 3b was replotted as histograms to show expression of BLIMP1 in a and IRF4 in b respectively. **c,** Production of IgM in culture supernatants of cells cultured as in Fig 3a. **d,** Expression of mouse Ufbp1 (mUfbp1) and human Ufbp1 (hUfbp1) in $GFP^{+}CD138^{+}$ populations sorted from B cell cultures from indicated mice infected with specified retroviruses from Fig 3e was determined by RT-PCR. The gel images were inverted and therefore, DNA bands and background appear black and white respectively. **e**, Production of IgM in culture supernatants of sorted $GFP^{+}CD138^{+}$ cells from Fig 3e after overnight culture. Error bars represent mean ± SE. *** P < 0.001. Unpaired student's two-tailed t-test was used. A representative of at least two experiments is shown.

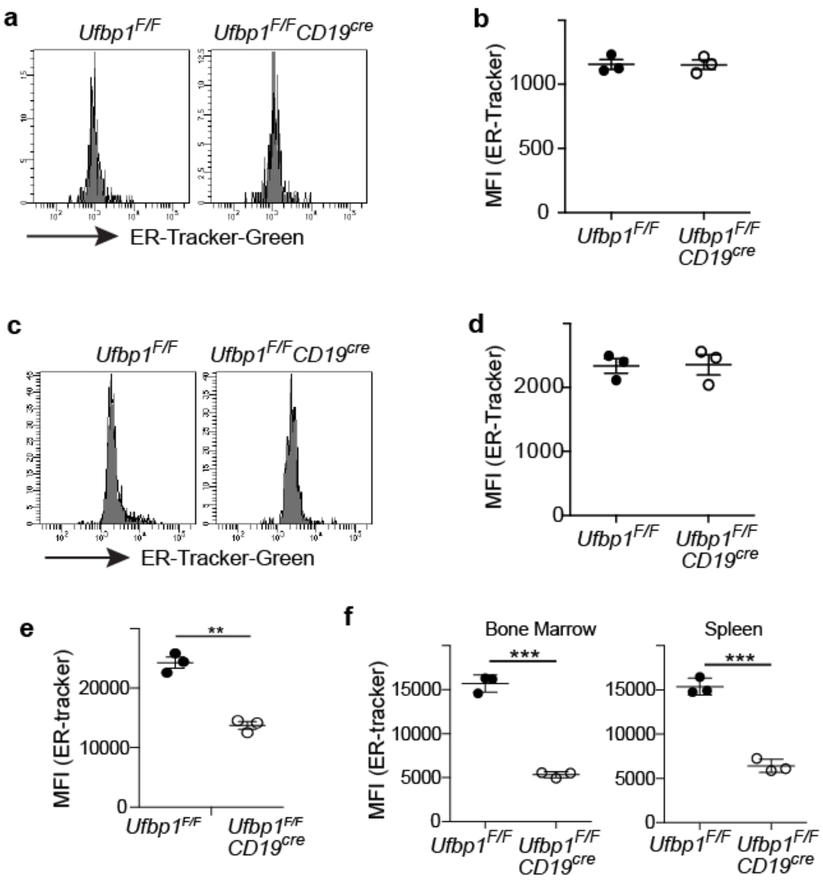

Supplementary Figure 4. Ufbp1 regulates ER mass in plasma cells. a and c, Naïve B cells were cultured with LPS. Flow cytometry was used to detect staining for ER-tracker on total cells on day 1 in a and day 2 in c. **b and d,** Quantification of ER-tracker staining in a and c respectively. **e**, naïve B cells were cultured with LPS, IL-4 and IL-5 as in Fig 3a. On day 4, ER-tracker staining on plasmablasts ($CD138^+TACI^+$ cells) was quantified by flow cytometry. **f,** Quantification of ER-tracker staining in plasma cells ($CD138^+TACI^+$ cells) by flow cytometry from bone marrow and spleens of indicated mice. Error bars represent mean ± SE (n= 3 mice/group). ** $P < 0.01$, *** $P < 0.001$. Unpaired student's two-tailed t-test was used. A representative of at least two experiments is shown.

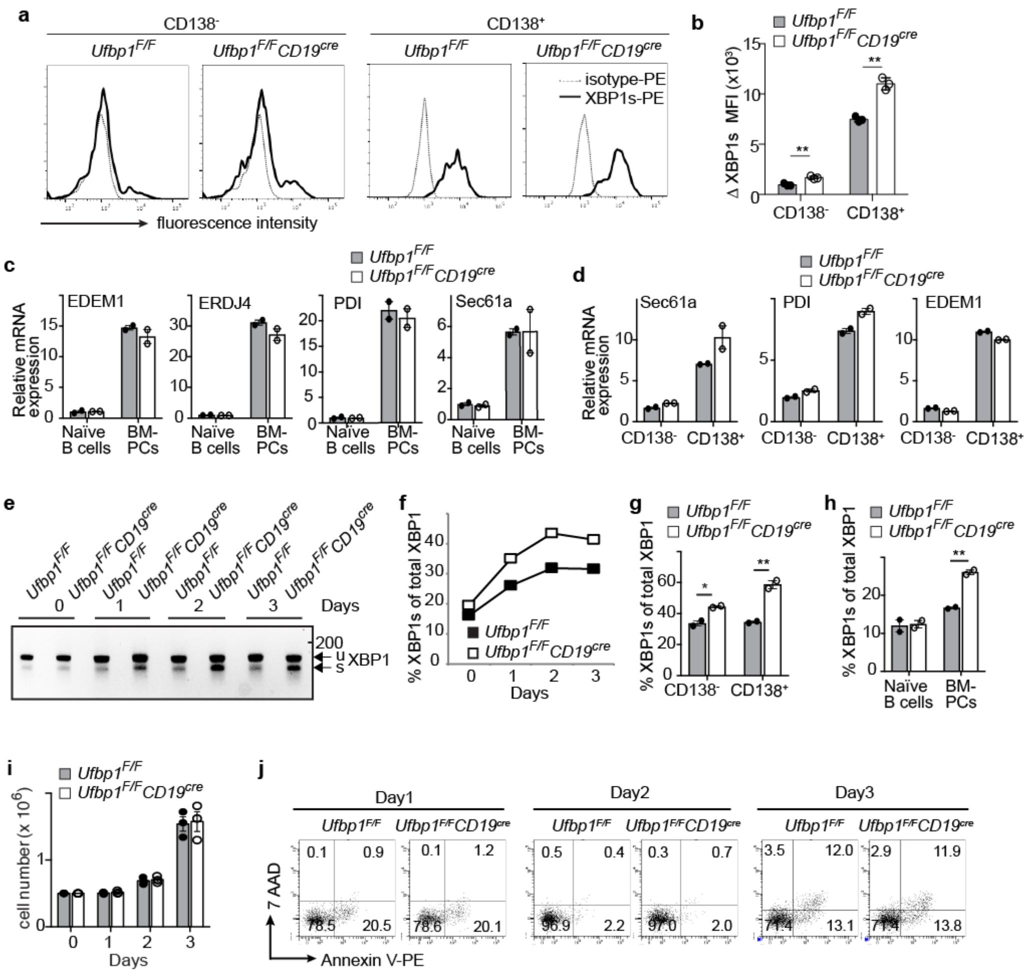

Supplementary Figure 5. XBP1 splicing in Ufbp1-deficient ASCs. a, Naïve B cells from *Ufbp1*$^{F/F}$ and *Ufbp1*$^{F/F}$*CD19*^{cre} mice were stimulated with LPS, IL-4 and IL-5 as in Fig 3a. Four days later, XBP1s staining in CD138^{-} and CD138^{+} cells were evaluated by flow cytometry. ΔMFI represents the difference between anti-XBP1s and control isotype IgG1 staining. **b,** Quantification of XBP1s staining in a. **c,** Expression of EDEM1, ERDJ4, PDI and Sec61 normalized against β-actin in naïve B cells and bone marrow plasma cells from indicated mice was quantified by qRT-PCR. **d,** Expression of Sec61, PDI and EDEM1 normalized against β-actin in CD138^{-} and CD138^{+} cells sorted from LPS-stimulated B cell cultures was quantified by qRT-PCR. **e,** Naïve B cells from indicated were cultured with LPS. Total XBP1 (unspliced and spliced) was detected by RT-PCR at indicated time points. The gel image was inverted and therefore, DNA bands and background appear black and white respectively. **f,** Quantification of XBP1 splicing in e. **g,** Quantification of XBP1 splicing in CD138^{-} and CD138^{+} cells obtained as in d. **h,** Quantification of XBP1 splicing in naïve B cells and bone marrow plasma cells of indicated mice. **i,** Naïve B cells (0.5 x 10^6/well in 24 well plate) from *Ufbp1*$^{F/F}$ and *Ufbp1*$^{F/F}$*CD19*^{cre} mice were cultured with LPS. The numbers of live cells recovered at indicated time points are shown. **j,** LPS-stimulated B cells from *Ufbp1*$^{F/F}$ and *Ufbp1*$^{F/F}$*CD19*^{cre} mice were analyzed for annexin-V and 7-AAD staining by flow cytometry on indicated day of culture. Error bars represent mean ± SE. *P < 0.05, ** P < 0.01. Unpaired student's two-tailed t-test was used. A representative of at least two experiments is shown.

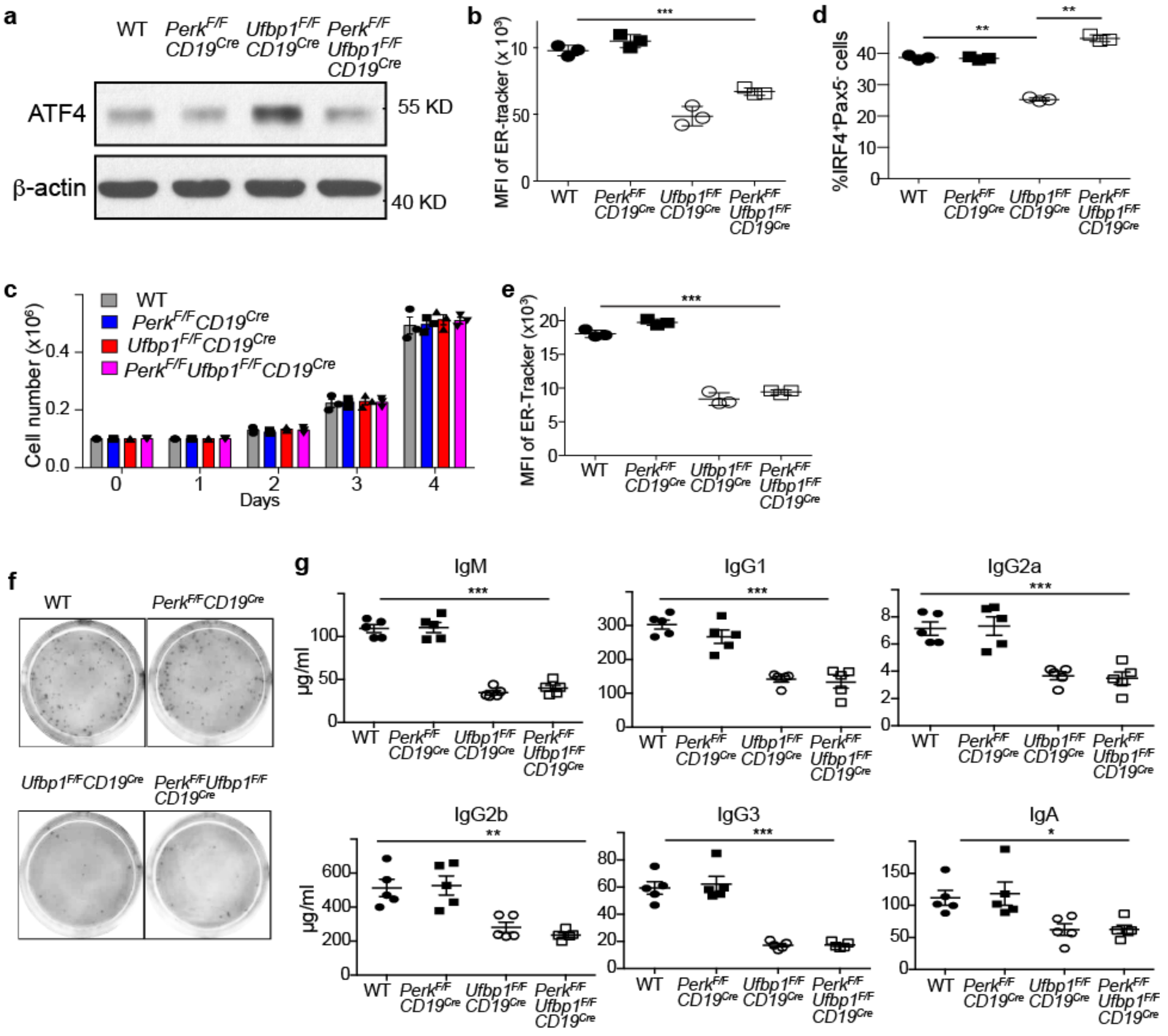

Supplementary Figure 6. Defective Ig production by Ufbp1-deficient ASCs is PERK-independent. **a,** naïve B cells from indicated mice were activated with LPS. On day 1, total cell lysates were immunoblotted with ATF4 and β-actin. **b,** Quantification of ER-tracker staining of bone marrow plasma cells ($CD138^+TACI^+$ cells) by flow cytometry from indicated mice. **c,** Naïve B cells (0.1 x 10^6) from indicated mice were cultured with LPS, IL-4 and IL-5. The number of live cells recovered at indicated time points are shown. **d,** Naïve B cells from indicated mice were cultured as in c. On day 4, frequencies of $IRF4^+Pax5^-$ cells was quantified by flow cytometry. **e**, Naïve B cells from indicated mice were cultured as in c. On day 4, ER tracker staining on plasmablasts ($CD138^+TACI^+$ cells) was quantified by flow cytometry. **f**, Representative ELISpot wells showing immunoglobulin secretion by LPS-induced plasmablasts from indicated mice. **g**, Presence of indicated immunoglobulin isotypes in the serum of indicated mice was quantified using ELISA (n=5 mice/genotype). Error bars represent mean ± SE. *$P < 0.05$, ** $P < 0.01$, *** $P < 0.001$. Unpaired student's two-tailed t-test was used. A representative of two experiments is shown.

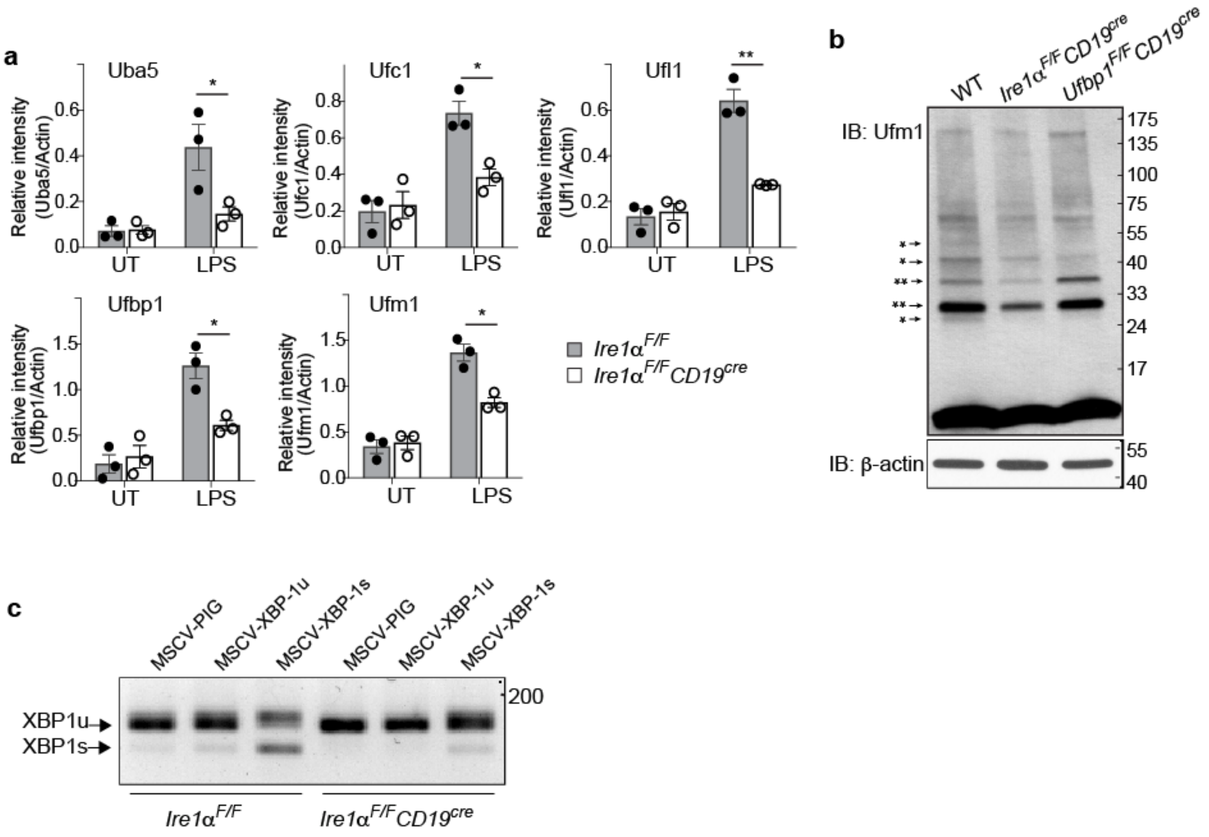

Supplementary Figure 7. Role of IRE1/XBP1 axis in regulation of Ufbp1.
a, Quantification of intensity of the indicated molecules normalized against β-actin in Fig 7b. The error bars represent mean ± SE of 3 experiments. * $P < 0.05$, ** $P < 0.01$. Unpaired student's two-tailed t-test was used. **b,** Naïve B cells from WT, *Ire1*$\alpha^{F/F}$*CD19*^{cre} and *Ufbp1*$^{F/F}$*CD19*^{cre} mice were cultured with LPS. On day three total cell lysates of cells were prepared and immunoblotted with antibody against Ufm1 and β-actin. * indicates the bands with decreased intensity in both *Ire1*$\alpha^{F/F}$*CD19*^{cre} and *Ufbp1*$^{F/F}$*CD19*^{cre} cells than WT cells. ** indicates the bands with decreased intensity in *Ire1*$\alpha^{F/F}$*CD19*^{cre} cells than WT cells. **c,** Expression of XBP1s and XBP1u in GFP^{+} cells from Fig 7d. The gel image was inverted and therefore, DNA bands and background appear black and white respectively. A representative of at least two experiments is shown.

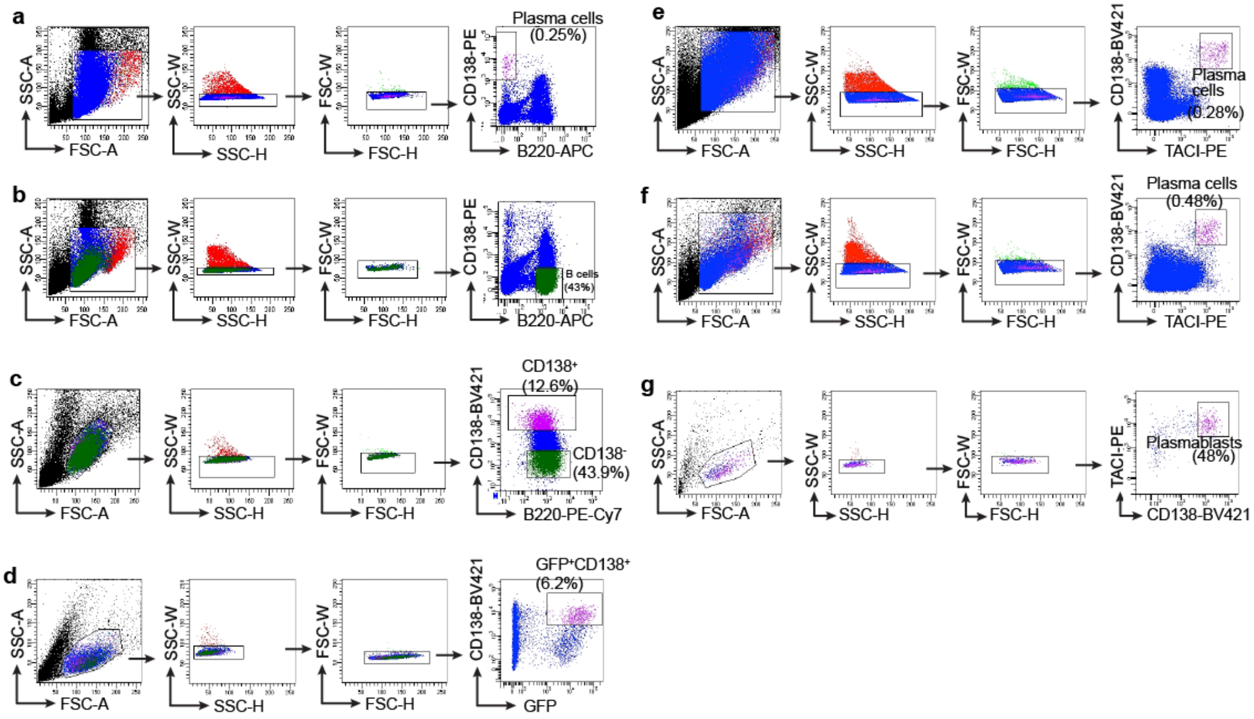

Supplementary Figure 8. Flow cytometric sorting strategy. Flow cytometric gating strategy for cell sorting. (**a**) Gating strategy to sort bone marrow plasma cells used for experiments presented in Figs 4f, 6k, 7a, Supplementary Figs 2a and 5c, h. (**b**) Gating strategy to sort naive B cells used for experiments presented in Fig 7a, Supplementary Figs 2a and 5c, h. (**c**) Gating strategy to sort CD138^{-} and/or CD138^{+} cells from LPS-stimulated B cell cultures for experiments presented in Figs 4e, 6i-j, l, 7c-d, Supplementary Figs 5d, g, 6f and 7c. (**d**) Gating strategy to sort GFP^{+}CD138^{+} cells from LPS, IL-4 and IL-5 stimulated B cells cultures transduced with retroviruses. GFP expression marks the cells transduced with retroviruses. These cells were used in Supplementary Figs 3d-e. (**e**) Gating strategy for identification of bone marrow plasma cells (CD138^{+}TACI^{+}) for ER-tracker staining in Supplementary Figs 4f and 6b. (**f**) Gating strategy for identification of splenic plasma cells (CD138^{+}TACI^{+}) for ER-tracker staining in Supplementary Figs 4f, 6g and 6h. (**g**) Gating strategy for identification of plasmablasts (CD138^{+}TACI^{+}) for ER-tracker staining in Supplementary Figs 4e and 6e.

Supplementary Figure 9. Original Full Western blots. In some experiments transferred membrane was cut and probed with appropriate antibodies. Please note that anti-Ufm1 immunoblot in Supplementary Fig 7b shows the full western blot.

Figure 4a

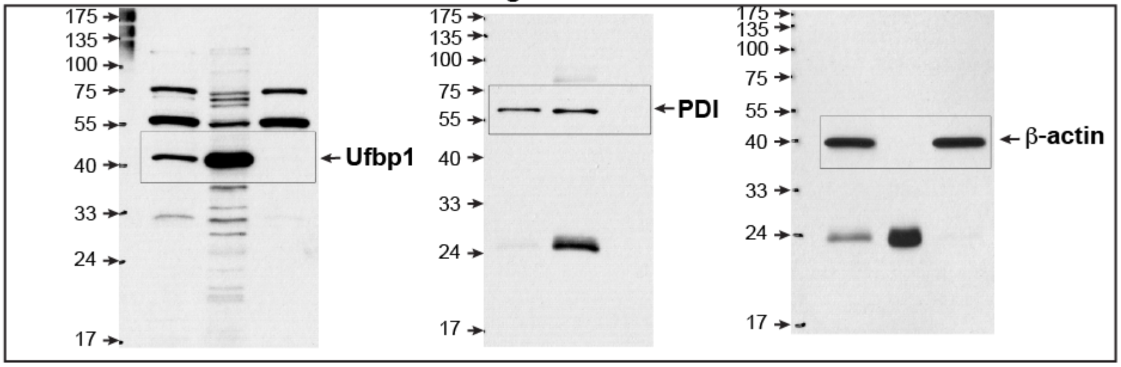

Figure 5c

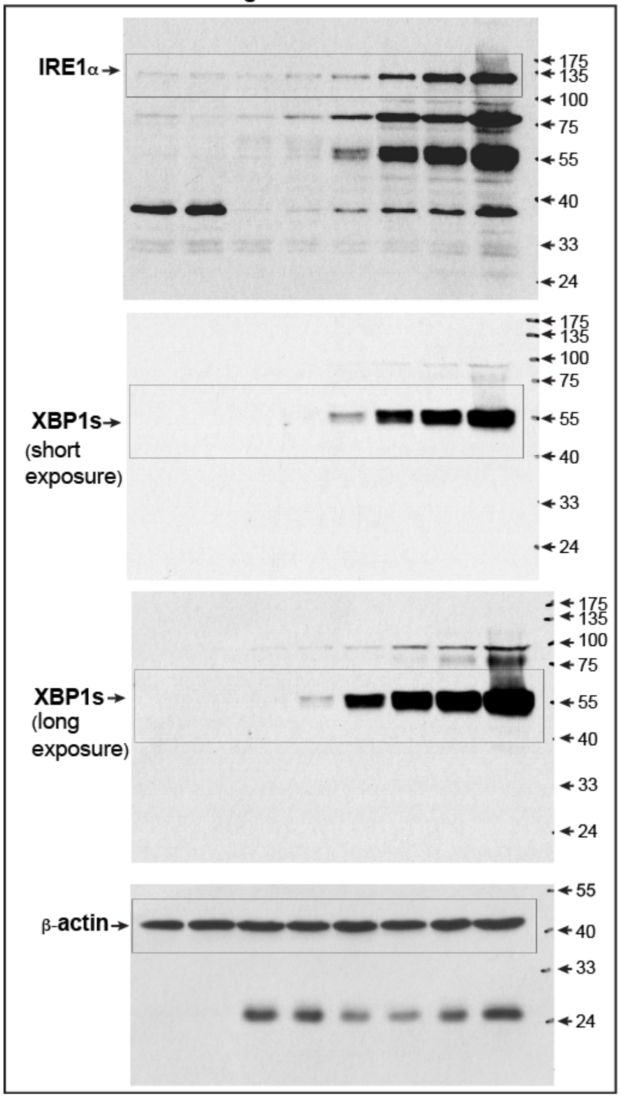

Figure 5f

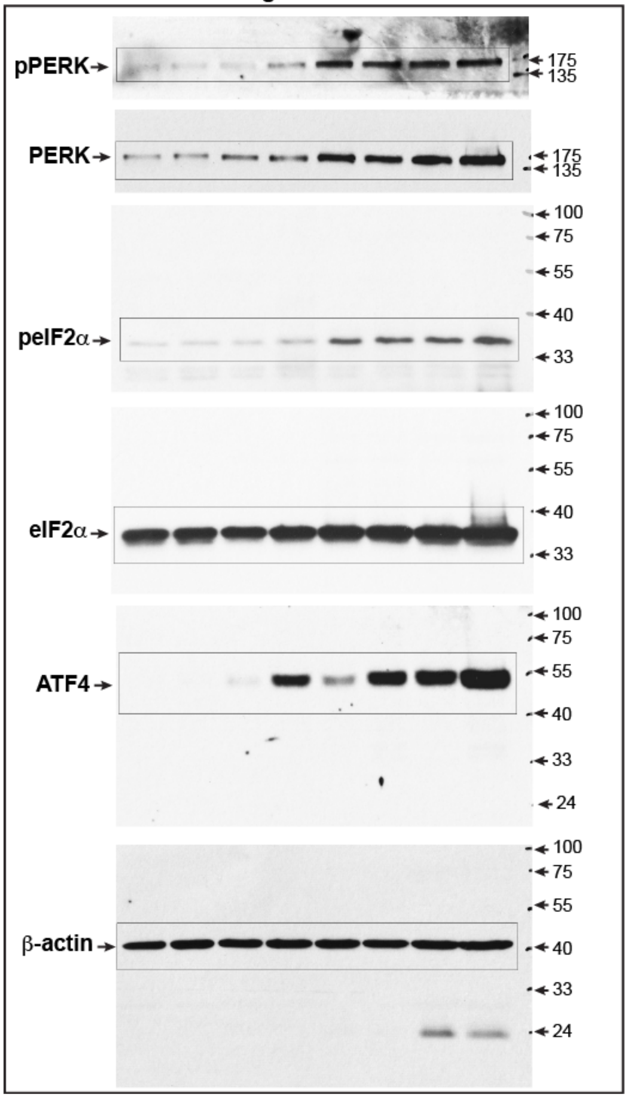

Figure 7b

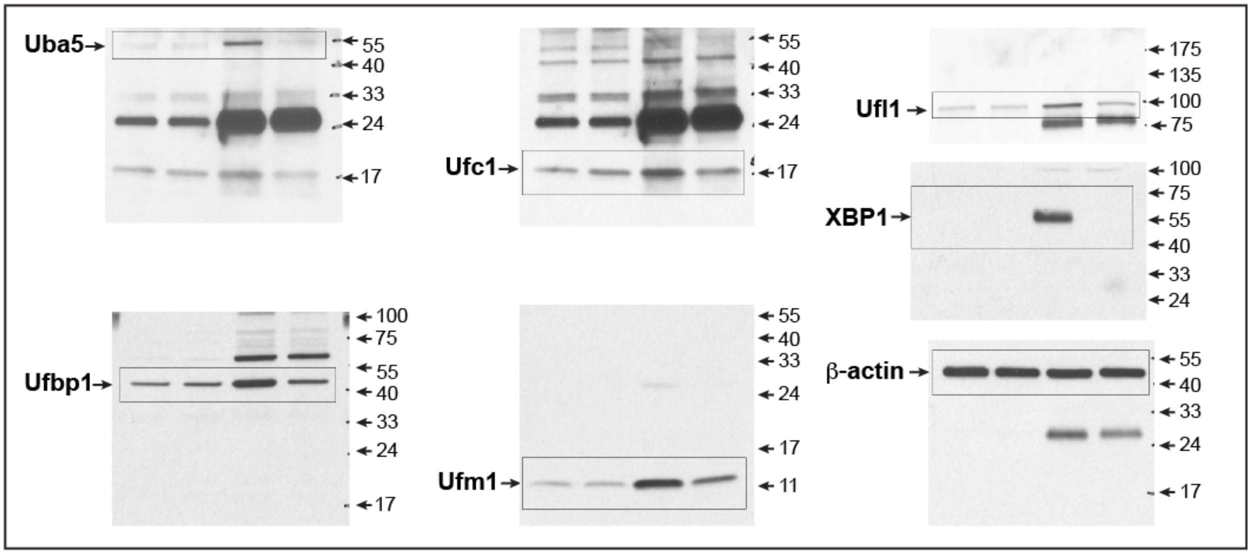

Supplementary Figure 1f

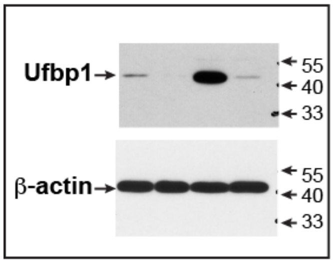

Supplementary Figure 6a

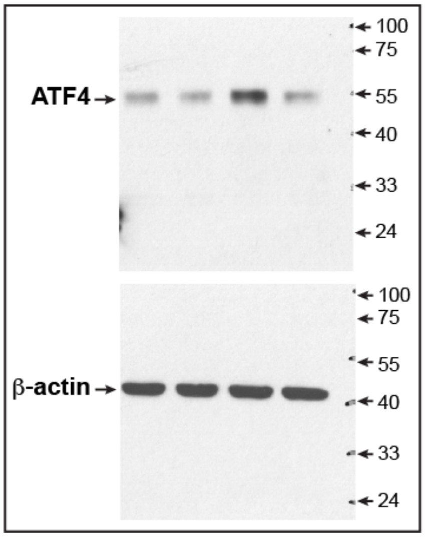

Supplementary Figure 7b

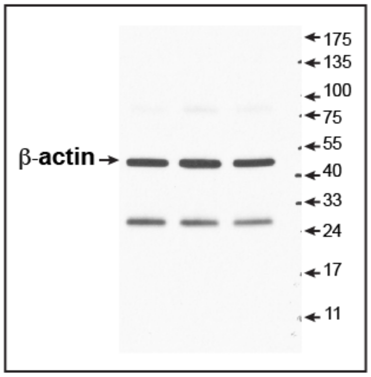

Supplementary Table 1. Sequences of PCR primers used in this study

Gene	Forward Primer	Reverse Primer
Uba5	CAA GCT ATG TTC ACG GCA GA	AGT TGT TTT GCC CAC CAC TC
Ufc1	AAA TTG CAG TCC CTG AGC TG	GTG CTG GAT CAC ACC CTT CT
Ufl1	ACA CGG TTG TGG TGA GTG AA	GTT GCT TTC TTC CTG CGT TC
mUfbp1	GAA GCC AGC AGA AGT TCA CC	GAA GCC GTT CCT CTT CCT TC
Ufm1	CCG TTC ACA GCA GTG CTA AA	CTG AAA GGC TGT GTG TTC CA
Ire1α	ACC TGA AGC CCC ACA ACA TT	AGC TTC TTG CAG AGG CCA AA
Edem1	AAG CCC TCT GGA ACT TGC G	AAC CCA ATG GCC TGT CTG G
Erdj4	TAA AAG CCC TGA TGC TGA AGC	TCC GAC TAT TGG CAT CCG A
Pdi	CAA GAT CAA GCC CCA CCT GAT	AGT TCG CCC CAA CAA GTA CTT
Sec61a	CTA TTT CCA GGG CTT CCG AGT	AGG TGT TGT ACT GGC CTC GGT
Xbp1	ACA CGC TTG GGA ATG GAC AC	CCA BTG GGA AGA TGT TCT GGG
hUfbp1	ACAGTCCCAGAGCTTCCTGA	TGCGATTTATGGTGTCCTGA
β-actin	GGC TGT ATT CCC CTC CAT CG	CCA GTT GGT AAC AAT GCC ATG T
