# Supplementary material for: Ufbp1 promotes plasma cell development and ER expansion by modulating distinct branches of UPR
Source: Nat Commun. 2019 Mar 6;10:1084. doi: 10.1038/s41467-019-08908-5 (PMC6403283; doi:10.1038/s41467-019-08908-5)
Supplement: Supplementary file 3 — Reporting Summary [file 41467_2019_8908_MOESM3_ESM.pdf]

nature research

Corresponding author(s): Nagendra Singh

Last updated by author(s): Jan 14, 2019

Reporting Summary

Nature Research wishes to improve the reproducibility of the work that we publish. This form provides structure for consistency and transparency in reporting. For further information on Nature Research policies, see Authors & Referees and the Editorial Policy Checklist.

Statistics

For all statistical analyses, confirm that the following items are present in the figure legend, table legend, main text, or Methods section.

n/a	Confirmed	
☐	☒	The exact sample size (n) for each experimental group/condition, given as a discrete number and unit of measurement
☐	☒	A statement on whether measurements were taken from distinct samples or whether the same sample was measured repeatedly
☐	☒	The statistical test(s) used AND whether they are one- or two-sided<br>*Only common tests should be described solely by name; describe more complex techniques in the Methods section.*
☒	☐	A description of all covariates tested
☐	☒	A description of any assumptions or corrections, such as tests of normality and adjustment for multiple comparisons
☐	☒	A full description of the statistical parameters including central tendency (e.g. means) or other basic estimates (e.g. regression coefficient) AND variation (e.g. standard deviation) or associated estimates of uncertainty (e.g. confidence intervals)
☐	☒	For null hypothesis testing, the test statistic (e.g. F, t, r) with confidence intervals, effect sizes, degrees of freedom and P value noted<br>*Give P values as exact values whenever suitable.*
☒	☐	For Bayesian analysis, information on the choice of priors and Markov chain Monte Carlo settings
☒	☐	For hierarchical and complex designs, identification of the appropriate level for tests and full reporting of outcomes
☒	☐	Estimates of effect sizes (e.g. Cohen's d, Pearson's r), indicating how they were calculated

Our web collection on statistics for biologists contains articles on many of the points above.

Software and code

Policy information about availability of computer code

Data collection	Flow Cytometry: FACSDIVA V8, Transmission electron microscopy: DigitalMicrograph 2.1.1, Elisa reader: Gen 5 2.09, ELISpot: , CTL switchboard 2.5.4, qPCR: Stepone Software V2.2, Microscopy: BZ-x Viewer
Data analysis	FACSDIVA V8 and Flow Jo V10.5.3 for analysis of flow cytometry data, Microsoft excel, Stepone Software V2.2 for analysis of qPCR data, Adobe Photoshop CS4 for quantification of western blots. Graph Pad Prism

For manuscripts utilizing custom algorithms or software that are central to the research but not yet described in published literature, software must be made available to editors/reviewers. We strongly encourage code deposition in a community repository (e.g. GitHub). See the Nature Research guidelines for submitting code & software for further information.

Data

Policy information about availability of data

All manuscripts must include a data availability statement. This statement should provide the following information, where applicable:

- Accession codes, unique identifiers, or web links for publicly available datasets
- A list of figures that have associated raw data
- A description of any restrictions on data availability

There is no restriction on availability of data

Field-specific reporting

Please select the one below that is the best fit for your research. If you are not sure, read the appropriate sections before making your selection.

☒ Life sciences ☐ Behavioural & social sciences ☐ Ecological, evolutionary & environmental sciences

Life sciences study design

All studies must disclose on these points even when the disclosure is negative.

Sample size	3-5 animals/group were used used for all the experiments. This was sufficient to find statistical significance.
Data exclusions	No data were excluded
Replication	Each experiment was repeated at least 2 times and results were reproducible
Randomization	Age-matched male and female mice were randomly assigned to experiments
Blinding	Investigators were not blinded during data collection or analysis

Reporting for specific materials, systems and methods

We require information from authors about some types of materials, experimental systems and methods used in many studies. Here, indicate whether each material, system or method listed is relevant to your study. If you are not sure if a list item applies to your research, read the appropriate section before selecting a response.

Materials & experimental systems

n/a	Involved in the study	
☐	☒	Antibodies
☐	☒	Eukaryotic cell lines
☒	☐	Palaeontology
☐	☒	Animals and other organisms
☒	☐	Human research participants
☒	☐	Clinical data

Methods

n/a	Involved in the study	
☒	☐	ChIP-seq
☐	☒	Flow cytometry
☒	☐	MRI-based neuroimaging

Antibodies

Antibodies used

Flow cytometry antibodies:
CD4-PerCP-CY5.5 (Clone RM4-5, ThermoFisher cat#45-0042-80, Lot: E08291-1632), CD8-PerCP-CY5.5 (Clone 53-6.7, ThermoFisher cat#45-0081-80, Lot: E08299-1631), CD19-PerCP-CY5.5 (Clone 6D5, Biolegend cat#115534, Lot: B183173), B220-PE-Cy7 (Clone RA3-6B2, Biolegend cat#103222, Lot: B210434), CD11b-PerCP-CY5.5 (clone: M1/70, ThermoFisher, cat # 45-0112-80, Lot: E08303-1633), B220-APC (Clone RA3-6B2, BD Biosciences, cat#553092, Lot: 5139848), CD138-PE (Clone 281-2, BD Biosciences cat#562610, Lot: 5195835), CD138-BV421 (Clone 281-2, BD Biosciences cat# cat#562610, Lot: 7061869), CD138-PE (Clone 281-2, BD Biosciences cat# cat#561070, Lot: 5195835), IgD-FITC (Clone 11-26c.2a, BD Biosciences, cat#553439, Lot: 4199925) IgD-AlexaFluor700 (Clone 11-26c.2a, Biolegend cat#405729, Lot: B225769), IgM-BV786 (Clone R6-60.2, BD Biosciences cat#564028, Lot: 5079987), Gr1-PerCP-CY5.5 (Clone RB6-8C5, BD Biosciences cat#561103, Lot: 3123803), BLIMP1-AlexaFluor647 (Clone 5E7, Biolegend, cat#150004, Lot: B271830), IRF4-PE (Clone 3E4, ThermoFisher cat#12-9858-80, Lot: 4303084), XBP-1s-PE (Clone Q3-695, BD Biosciences cat#562642, Lot: 4220630), Pax5- PerCP-Cy5.5 (Clone 1H9, Biolegend, cat#649709, Lot: B222379), TACI-PE (Clone 8F10, Biolegend, cat#133403, Lot: B262672)

Western blot and Immunofluorescence antibodies
UFBP1 (Proteintech, Cat#21445-1-AP, Lot: 00014958), b-ACTIN (Clone AC-15, Sigma-Aldrich Cat#A5441, Lot: 030M4788), , PDI (Clone: 34/PDI, BD Biosciences), IRE1α (Clone 14C10, Cell signaling Cat#3294P, Lot:9), pPERK (Clone T980, Cell signaling Cat#3179, Lot:19), PERK (Clone C33E10, Cell signaling Cat#3192, Lot:9), XBP-1s (Clone Poly6195, Biolegend Cat#619502, Lot: B109131), peIF2α (Clone D9G8, Cell signaling Cat#3398, Lot:2), eIF2α (Clone D7D3, Cell signaling Cat#5324, Lot:1), ATF4 (Clone D4B8, Cell signaling Cat#11815, Lot:2), Ufm1 (Clone EPR4264(2)) Abcam Cat#ab109305, Lot: GR298180-5),Goat anti-Rabbit IgG-HRP (Jackson ImmunoResearch, cat # 111-035-003), Goat anti-Mouse IgG-HRP (Jackson ImmunoResearch, cat # 115-035-003), Goat anti-Rat IgG-HRP (Jackson ImmunoResearch, cat # 112-035-003), Goat anti-Rabbit IgG-AlexaFluor488 (Jackson ImmunoResearch, cat # 111-545-144), Goat anti-Mouse IgG-AlexaFluor594 (Jackson ImmunoResearch, cat # 115-585-146).

Antibodies against Uba5, UFC, and Ufl1 were obtained from Dr. Honglin Li laboratory (Augusta University), have been published previously (Cai et al., 2015, PLoS genetics 11, e1005643, Zhang et al., 2012, PLoS One 7, e48587, Wu et al., J. Biol Chem 285, 15126–15136)

Validation

Following antibodies have been routinely used by multiple publication and validation data is also available on manufacturer's website
CD4 (Clone RM4-5, ThermoFisher cat#45-0042-80), CD8 (Clone 53-6.7, ThermoFisher cat#45-0081-80), CD11b-PerCP-CY5.5 (clone: M1/70, ThermoFisher, cat # 45-0112-80), CD19 (Clone 6D5, Biolegend cat#115534), B220 (Clone RA3-6B2, Biolegend cat#103222), CD79b (Clone HM79-12, Biolegend cat#132805), CD138 (Clone 281-2, BD Biosciences cat#562610), IgD (Clone

11-26c.2a, Biolegend cat#405729), IgM (Clone R6-60.2, BD Biosciences cat#564028), GL7 (Clone GL7, Biolegend cat#144605), Gr1 (Clone RB6-8C5, BD Biosciences cat#561103), BLIMP1 (Clone 5E7, Biolegend cat#150004), IRF4 (Clone 3E4, ThermoFisher cat#12-9858-80), XBP-1s (Clone Q3-695, BD Biosciences cat#562642), TACI (Clone 8F10, Biolegend, cat#133403)
b-ACTIN (Clone AC-15, Sigma-Aldrich Cat#A5441), PDI (Clone: 34/PDI, BD Biosciences), IRE1α (Clone 14C10, Cell signaling Cat#3294P), pPERK (Clone T980, Cell signaling Cat#3179), PERK (Clone C33E10, Cell signaling Cat#3192), XBP-1s (Clone Poly6195, Biolegend Cat#619502), peIF2α (Clone D9G8, Cell signaling Cat#3398), eIF2α (Clone D7D3, Cell signaling Cat#5324), ATF4 (Clone D4B8, Cell signaling Cat#11815), Ufm1 (Clone EPR4264(2)) Abcam Cat#ab109305), Goat anti-Rabbit IgG-HRP (Jackson ImmunoResearch, cat # 111-035-003), Goat anti-Mouse IgG-HRP (Jackson ImmunoResearch, cat # 115-035-003), Goat anti-Rat IgG-HRP (Jackson ImmunoResearch, cat # 112-035-003), Goat anti-Rabbit IgG-AlexaFluor488 (Jackson ImmunoResearch, cat # 111-545-144), Goat anti-Mouse IgG-AlexaFluor594 (Jackson ImmunoResearch, cat # 115-585-146).
Antibodies against Uba5, UFC, and Ufl1 were obtained from Dr. Honglin Li laboratory (Augusta University), have been published previously (Cai et al., 2015, PLoS genetics 11, e1005643, Zhang et al., 2012, PLoS One 7, e48587, Wu et al., J. Biol Chem 285, 15126–15136)

Eukaryotic cell lines

Policy information about cell lines

Cell line source(s)	Plat-E from Cell Biolabs
Authentication	Plat-E cell from Cell Biolabs was used for packaging of retroviruses. These cells are rigorously tested and authenticated by Cell Biolabs for production of retroviruses. Plat-E has been used by several studies for production of retroviruses. Some of these publications are available on manufacturers website
Mycoplasma contamination	cells were negative for mycoplasma
Commonly misidentified lines (See ICLAC register)	Plat-E was not listed in the most recent ICLAC list

Animals and other organisms

Policy information about studies involving animals; ARRIVE guidelines recommended for reporting animal research

Laboratory animals	C57BL/6, Perkf/f and CD19Cre were obtained from Jackson laboratory. Ufbp1f/f mice and Ire1af/f mice have been described (Cai et al., PLoS genetics 11, e1005643, 2015, Zhang et al., he EMBO journal 30, 1357-1375, 2011). Ufbp1f/f, Perkf/f and Ire1af/f mice were crossed with CD19Cre mice to obtain Ufbp1f/fCD19cre, Perkf/fCD19cre and Ire1af/fCD19cre mice respectively. Mice of both sexes between 6-24 weeks of age were randomly used. The Institutional Animal Care and Use Committee (IACUC), Augusta University approved all the animal procedures.
Wild animals	*Provide details on animals observed in or captured in the field; report species, sex and age where possible. Describe how animals were caught and transported and what happened to captive animals after the study (if killed, explain why and describe method; if released, say where and when) OR state that the study did not involve wild animals.*
Field-collected samples	*For laboratory work with field-collected samples, describe all relevant parameters such as housing, maintenance, temperature, photoperiod and end-of-experiment protocol OR state that the study did not involve samples collected from the field.*
Ethics oversight	*Identify the organization(s) that approved or provided guidance on the study protocol, OR state that no ethical approval or guidance was required and explain why not.*

Note that full information on the approval of the study protocol must also be provided in the manuscript.

Flow Cytometry

Plots

Confirm that:

☒ The axis labels state the marker and fluorochrome used (e.g. CD4-FITC).

☒ The axis scales are clearly visible. Include numbers along axes only for bottom left plot of group (a 'group' is an analysis of identical markers).

☐ All plots are contour plots with outliers or pseudocolor plots.

☒ A numerical value for number of cells or percentage (with statistics) is provided.

Methodology

Sample preparation	Single cell suspension from spleen, lymph nodes and bone marrow were prepared. Red blood cells were lysed and cells were filtered through 70 µm fileter. Cells were stained with appropriate antibodies (diluted in PBS, 1% FBS and 10 mM Hepes pH7.4) or dyes (in manufacturer recommended buffer) and analyzed. Where indicated, LPS-stimulated B cells were stained and analyzed in a similar way.
Instrument	Data were collected and analyzed on BD LSRII analyzer

Software	FACSDIVA V8 and FlowJo were used for data collection and analysis
Cell population abundance	Post sort purity was >95%.
Gating strategy	Gating strategy is provided in Supplementary Figure 8.

☒ Tick this box to confirm that a figure exemplifying the gating strategy is provided in the Supplementary Information.
